# Large-Scale Social Media Analysis Reveals Emotions Associated with Nonmedical Prescription Drug Use

**DOI:** 10.34133/2022/9851989

**Published:** 2022-04-27

**Authors:** Mohammed Ali Al-Garadi, Yuan-Chi Yang, Yuting Guo, Sangmi Kim, Jennifer S. Love, Jeanmarie Perrone, Abeed Sarker

**Affiliations:** ^1^Department of Biomedical Informatics, School of Medicine, Emory University, Atlanta, GA, USA; ^2^Department of Computer Science, Emory University, Atlanta, GA, USA; ^3^School of Nursing, Emory University, Atlanta, GA, USA; ^4^Department of Emergency Medicine, School of Medicine, Oregon Health & Science University, Portland, OR, USA; ^5^Department of Emergency Medicine, Perelman School of Medicine, University of Pennsylvania, Philadelphia, PA, USA; ^6^Department of Biomedical Engineering, Georgia Institute of Technology and Emory University, Atlanta, GA, USA

## Abstract

*Background.* The behaviors and emotions associated with and reasons for nonmedical prescription drug use (NMPDU) are not well-captured through traditional instruments such as surveys and insurance claims. Publicly available NMPDU-related posts on social media can potentially be leveraged to study these aspects unobtrusively and at scale.*Methods.* We applied a machine learning classifier to detect self-reports of NMPDU on Twitter and extracted all public posts of the associated users. We analyzed approximately 137 million posts from 87,718 Twitter users in terms of expressed emotions, sentiments, concerns, and possible reasons for NMPDU via natural language processing.*Results.* Users in the NMPDU group express more negative emotions and less positive emotions, more concerns about family, the past, and body, and less concerns related to work, leisure, home, money, religion, health, and achievement compared to a control group (i.e., users who never reported NMPDU). NMPDU posts tend to be highly polarized, indicating potential emotional triggers. Gender-specific analyses show that female users in the NMPDU group express more content related to positive emotions, anticipation, sadness, joy, concerns about family, friends, home, health, and the past, and less about anger than males. The findings are consistent across distinct prescription drug categories (opioids, benzodiazepines, stimulants, and polysubstance).*Conclusion.* Our analyses of large-scale data show that substantial differences exist between the texts of the posts from users who self-report NMPDU on Twitter and those who do not, and between males and females who report NMPDU. Our findings can enrich our understanding of NMPDU and the population involved.

## 1. Introduction

Nonmedical prescription drug use (NMPDU) involves the use of prescription drugs without a prescription or for reasons other than what the drug was intended for by the prescriber [1]. NMPDU is an unremitting public health concern globally and in the United States (US) [2]. Commonly misused prescription drugs include but are not limited to opioids, central nervous system stimulants, and benzodiazepines [3, 4]. Increases in NMPDU over recent years have led to increased adverse health outcomes, including emergency department visits and overdose deaths [5]. In the US, more than 90,000 drug overdose deaths were recorded in 2020, many of which were caused by prescription drugs, often due to coingestion or polysubstance use [6, 7]. While studies have attempted to characterize the reasons for NMPDU [8, 9], little is known about the emotional status of the consumers at the time of NMPDU. Studies investigating the influence of NMPDU on mental health have been primarily conducted through surveys. NMPDU involving opioids have been shown to be strongly associated with psychiatric disorders [10] (data for the study was derived from the National Epidemiologic Survey on Alcohol and Related Conditions-III). Analysis of data from the National Survey on Drug Use and Health (NSDUH) revealed associations between opioid misuse and suicide-related risk factors, and that users involved in NMPDU of opioids were at higher risk of suicidality and suicidal ideation [11, 12] compared to those who never used these medications nonmedically. Past studies [3, 13] supported similar findings and showed associations between NMPDU of opioids and major depressive disorder or depressive symptoms.

Survey-based studies about NMPDU face several obstacles related to data collection, such as slow collection rates, high costs, and limited sample sizes. Importantly, studies using surveys are unable to capture naturally occurring emotions due to experimental or instrumental manipulations that could introduce measurement and observation biases [14]. Social media can address some of the shortcomings of such traditional survey-based studies. Social media presents a unique opportunity to collect information related to NMPDU for analysis at a large scale discreetly and unobtrusively so that the users’ expressions are not manipulated by experimental settings or processes. Also, the rising popularity of social media platforms has resulted in tremendous growth in the public sharing of information. Publicly available, user-generated social media data contain naturally occurring communication phenomena describing users’ daily activities, issues, and concerns, which enable the execution of observational studies to understand social dynamics [15– 17] and human behaviors at the macrolevel, including behaviors related to NMPDU [18]. Indeed, past research has shown that social media users often share information about NMPDU publicly, which can be utilized for making macrolevel assessments of drug abuse-/misuse-related behaviors [19– 21]. Recent studies [21– 23] validated the utility of social media as a platform for monitoring NMPDU. For instance, a qualitative assessment of the text content from Twitter on NMPDU (specifically, prescription opioids) delivered insights about the epidemic of use and misuse of PMs at specific times [22]. Multiple studies have suggested that although users engaging in NMPDU may not voluntarily report their nonmedical use to medical experts, their self-reports in social media are detectable [21, 24, 25], and these can potentially be used for public health surveillance. A critical review [18] concluded that social media big data could be an effective resource to comprehend, monitor, and intervene in drug misuses and addiction problems.

In addition to behaviors, emotion-related contents on social media provide important information about the users’ psychological and physical health [26]. Negative emotion words of higher magnitudes are associated with greater psychological distress and worse physical health, while high-magnitude positive emotion words are associated with higher well-being and better physical health [26]. Demographic information about users, such as gender, may also be inferred from social media for differential behavior analysis. For example, social media-based research has shown that males and females have differing emotional tendencies under different circumstances, and certain online activities of female users are more susceptible to emotional orientations [27]. Recognizing the gender differences in user behaviors is a significant factor in user modelling and human-computer interaction, and the differences were investigated in previous studies through the analyses of lexical contents, including emoticons [28– 30]. In the context of NMPDU, understanding gender differences between people who report NMPDU is particularly critical, as women specifically had often been underrepresented in past studies on the topic [31].

In this study, we sought to employ natural language processing (NLP) and machine learning approaches to study a large dataset from Twitter about three common prescription drug categories and their combinations (opioids, benzodiazepines, stimulants, and polysubstance—misuse of two or more different NMPDU category at the same time, typically referred to as coingestion) to investigate and answer the following main research questions: (i) How do the emotional contents expressed in the NMPDU groups’ Twitter profiles differ from those expressed in the non-NMPDU (control group) groups’ Twitter profiles? (ii) How do NMPDU tweets sentimentally differ from non-NMPDU tweets? And (iii) how do personal, social, biological, and core drive concerns expressed in the NMPDU groups’ Twitter profiles differ from those expressed in the non-NMPDU groups’ Twitter profiles? In addition to attempting to answer these questions, we use topic modeling on the NMPDU tweets to extract potential reasons for nonmedical use of each category of drugs, and we compare the distributions (of all the variables mentioned above) across males and females.

## 2. Methods

### 2.1. Data Collection

For NMPDU, similar to our previous study [32], which discussed designing a data-centric pipeline tool to collect NMPDU data from social media, we used a list of keywords (see Supplementary S.2) after consultation with the senior toxicology expert of our study (JP). We included a list of prescription drugs, including opioids, benzodiazepines, and central nervous system stimulants, which are known for their misuse/abuse potential. We also included people who reported NMPDU involving multiple drugs at the same time (polysubstance). First, we extracted approximately 3,287,703 tweets that contained at least one from a list of identified keywords related to prescription drugs from March 6, 2018, to January 14, 2020, to be the seeds for the collection of people who report NMPDU (NMPDU cohort or NMPDU users). We used an advanced NLP-based model (see NMPDU classification model) to classify the tweets automatically into one of four categories: NMPDU, consumption, mention, and unrelated. Mining NMPDU information from social media is more challenging than mining illicit drug use information, particularly because consumption of prescription drugs does not automatically indicate nonmedical use. We extracted the complete publicly available user profiles (i.e., all publicly available tweets) of users who posted the NMPDU tweets to build our experimental group (NMPDU users). We removed any user with less than 500 tweets. As shown in Table 1, we collected 49,833 NMPDU users with approximately 82 million tweets. For non-NMPDU users (control group), we randomly extracted publicly available profiles whose genders were reported in Liu and Ruths [33] and Volkova et al. [34], and who had not mentioned any identified prescription drug keywords in their profiles, resulting in 37,885 non-NMPDU users with approximately 55 million tweets. Overall, we included complete publicly available profiles of 87,718 users with approximately 137 million tweets. 

**Table 1:** Number of unique users and tweets in both NMPDU and non-NMPDU.

	Unique users	Number of tweets
Opioids	8,290	8,084,203
Stimulants	18,540	39,917,674
Benzodiazepines	14,773	23,085,707
Polysubstance	8,230	11,164,212

Total number of unique users and number of tweets		
Total NMPDU	49,833	82,251,796
Total non-NMPDU	37,885	55,352,960
Total	87,718	137,604,756

### 2.2. NMPDU Classification Model

We used an NLP text classification model developed and validated in our previous research [35] to distinguish NMPDU from non-NMPDU tweets. The model uses RoBERTa—a transformer-based language model—to classify tweets into (1) NMPDU (potential nonmedical use), (2) consumption (consumption but no evidence of nonmedical use), (3) mention (drug mentioned but no evidence of consumption), and (4) unrelated. Overall, the NMPDU classification model has an accuracy of 82.32%, and the $F_{1}$ scores for the classes are as follows: NMPDU 65%, consumption 91%, mention 88%, and unrelated 90%. 

### 2.3. Gender Label

The genders of the non-NMPDU users (control group) were released publicly on Twitter and reported in previous works [33, 34]. The gender distributions of the NMPDU users were estimated using an NLP text classification model described in the authors’ previous work [36]. This model uses users’ metadata (name, screen name, and description) and tweets to label the users using a binary gender paradigm (i.e., male and female) and has an accuracy of 94.4% on NMPDU users. 

### 2.4. Emotion Analysis

For emotion analysis, we used the word emotion lexicon curated by the National Research Council (NRC) of Canada [37]. The lexicon is a list of approximately 14,000 English words and their associations with eight basic emotions (anger, fear, anticipation, trust, surprise, sadness, joy, and disgust) according to Plutchik’s research on basic emotions [38]. The annotations were manually done by crowdsourcing [37]. The emotion lexicon has been used to study and categorize the emotion in the Twitter text by several prior studies [39– 41], and it is considered the benchmark for this domain of data. 

### 2.5. Sentiment Analyses of NMPDU Tweets (Sentiment Score Classifier)

We used VADER [42], an open-source Twitter sentiment model, which assigns numerical sentiment scores between +1 (extremely positive sentiment) and −1 (extremely negative sentiment) to each tweet. VADER has been used as the sentiment analyzer in several previous studies [14, 43]. Furthermore, a survey research [44] that compared the results of two classes of sentiment classifiers on four datasets from Twitter concluded that VADER has the best performance, with an overall accuracy of 99.04% (positive class: precision=99.16%, recall=99.16%, $F_{1}\text{score}=99.31$%; negative class: precision=98.77%, recall=98.12%, $F_{1}\text{score}=98.88$%). Even though VADER is optimized for social media data and has been shown to generate excellent results when applied to data from Twitter, misspellings and grammatical errors might impact the overall rating of the tweets. Moreover, tweets with certain concepts such as sarcasm might be rated by VADER incorrectly. 

### 2.6. Personal and Social Concern Analysis

We used the validated Linguistic Inquiry and Word Count (LIWC) [45] tool, which has been used to analyze several varieties of text, including social media text. The LIWC lexicon, which is designed to measure several behavioral and psychological dimensions from text, has been used in prior studies [39, 46, 47] for physiological measures of well-being analysis from social media. Examples of words related to each category are provided in supplementary document S.4 Table 5. A complete list of validated words in each category can be found in LIWC dictionary, which characterizes words into psychologically meaningful categories [45]. 

### 2.7. Statistical Testing

We used the Mann–Whitney U test, a nonparametric test, to compare outcomes between two independent groups. The Mann–Whitney U test examines whether two samples are possibly derived from the same population [48, 49]. The Mann–Whitney U test is used when the absence of normality distribution in both groups exists, and it compares the medians between the two populations. The histogram analysis and AD test results (see Supplementary S.3) confirmed the absence of normality distribution in both groups’ variables. Therefore, we used the nonparametric Mann–Whitney U test [48, 49] to compare the distributions. 

### 2.8. Topic Modeling

For topic modeling, we applied LDA [50], an effective unsupervised method that assumes that each document in a large dataset comprises subtopics represented by the words they contain. We initially cleaned the tweets by removing hyperlinks, digits, and stop words. Then, to decide the ideal number of topics for our model, we executed multiple models with different hyperparameter values (number of topics =5, 15, 20, 30, 40, and 50). We then inspected the word clusters in each set of subtopics and determined the most salient set of topics. Subsequently, we selected the 20-topic model. LDA was applied on tweets that were classified as positive, so we could assume that most of the tweets (as indicated by the classifier’s accuracy) represented nonmedical use. After the LDA model was executed, we presented the text segments identified to a domain expert included in the study who helped map these words into potential reasons. Therefore, the process of extracting potential reasons has an expert in the loop who was responsible for manually inspecting the frequent words within each category qualitatively, guided by the National Survey on Drug Use and Health (NSDUH) surveys [51]. We then qualitatively estimated the potential reasons for nonmedical uses for each category of NMPDU (see Supplementary S.5: Table 7,8,9,10). 

## 3. Results

### 3.1. NMPDU (Experimental Group) and Non-NMPDU (Control Group) Users

We included a total of 87,718 Twitter users and their >130 million posts in this study. To automatically characterize tweets (i.e., whether a tweet expresses self-reported NMPDU or not) mentioning specific medication keywords (see Supplementary S.2.1), we applied an automatic machine learning classifier, which was trained using a state-of-the-art NLP algorithm and a large manually annotated dataset. Table 1 presents the distribution of users and tweets in the NMPDU and non-NMPDU groups. 

### 3.2. Emotion Analysis

We investigated the emotion content differences in users’ tweets from the NMPDU and non-NMPDU groups (Table 2). We performed linguistic emotion analysis of the complete profile contents for both groups using the lexicon curated by the National Research Council (NRC), Canada, which contains a comprehensive list of approximately 14,182 English words related to anger, fear, anticipation, trust, surprise, sadness, joy, sentiment (negative and positive), and disgust [37]. We then used the Anderson–Darling (AD) test [52] and performed histogram analysis to check the normality distribution of the emotion-indicating variables in both groups. The histogram analysis and AD test results (see Supplementary S.3) confirmed the absence of normality distribution in all the emotion-indicating variables of both groups. Therefore, we used a nonparametric approach, the Mann–Whitney test [48, 49], to compare the distributions of emotion-indicating variables between users in the NMPDU and non-NMPDU groups. Table 2 presents the median Mann–Whitney U test results and the effect sizes of the comparisons between the NMPDU and control groups. It also presents comparisons between the NMPDU group and the control group for each medication category (i.e., opioids, benzodiazepines, stimulants, and polysubstance) in Supplementary S.4. 

**Table 2:** Comparison of emotional differences between users from the NMPDU and control groups.

Emotions	Median: NMPDU group	Median: (control group)	Different (NMPDU versus control) Mann–Whitney U test results	
			p value (p)	Effect sizes (r)
Positive	0.534	0.696	<0.001	0.40 ^**^
Trust	0.326	0.393	<0.001	0.28 ^*^
Anticipation	0.298	0.396	<0.001	0.44 ^**^
Fear	0.218	0.176	<0.001	0.31 ^**^
Anger	0.234	0.148	<0.001	0.56 ^***^
Negative	0.458	0.337	<0.001	0.45 ^**^
Sadness	0.215	0.169	<0.001	0.36 ^**^
Joy	0.284	0.368	<0.001	0.38 ^**^
Surprise	0.145	0.190	<0.001	0.39 ^**^
Disgust	0.196	0.113	<0.001	0.60 ^***^

Size effects (r): ^*^Small difference (0.1≤r<0.3). ^**^Moderate difference (0.3≤r<0.5). ^***^Large difference (0.5≤r<0.7). ^****^Very large difference (r≥0.7) [53].

The users from the NMPDU group tend to share significantly more content related to fear (p<0.001, r=0.31), anger (p<0.001, r=0.56), negative emotion (p<0.001, r=0.45), sadness (p<0.001, r=0.36), and disgust (p<0.001, r=0.60) compared to the users from the control group. The NMPDU users share significantly less content related to positive emotions (p<0.001, r=0.40), joy (p<0.001, r=0.38), trust (p<0.001, r=0.28), anticipation (p<0.001, r=0.44), and surprise (p<0.001, r=0.39) than the users from the control group (Table 2). These findings are consistent across all four medication categories considered in this study (Supplementary S.4, Table 4). 

#### 3.2.1. Gender Differences in Emotions within the NMPDU Group

**Figure 1:**
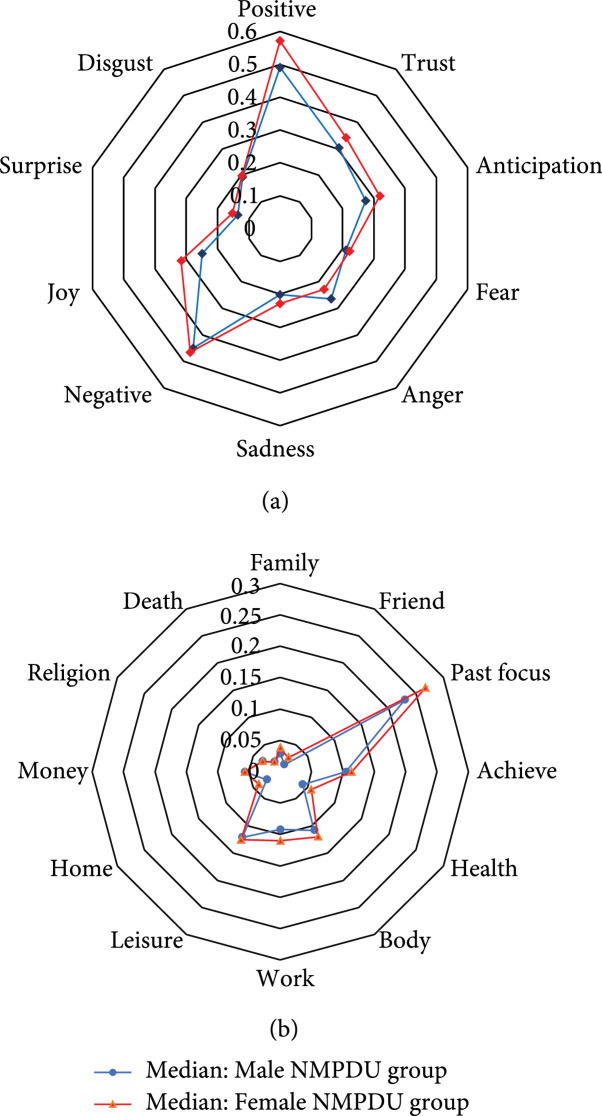
Summary comparison between the male and female users from the NMPDU group. (a) Emotional dimensions and (b) the personal and social concern content dimensions.

Within the NMPDU group, female users use more emotional content words/descriptors in the NMPDU-related social media posts compared to male users (Table 3 and Figure 1(a)). Specifically, female users express more content related to positive emotion (p<0.001,r=0.246), anticipation (p<0.001,r=0.247), sadness (p<0.001,r=0.21), and joy (p<0.001,r=0.38) compared to male users. In contrast, male users express significantly more content related to anger (p<0.001,r=0.07) than female users. The results also show no significant difference between males and females in content related to trust, fear, surprise, disgust, and negative emotions.

**Table 3:** Comparison of emotions between male and female users from the NMPDU.

Emotions	Median: Male NMPDU Group	Median: Female NMPDU Group	Different (male NMPDU versus female NMPDU) Mann–Whitney U test results	
			p value (p)	Effect sizes (r)
Positive	0.491	0.571	<0.001	0.246 ^*^
Trust	0.304	0.342	>0.001	—
Anticipation	0.274	0.319	<0.001	0.247 ^*^
Fear	0.212	0.223	>0.001	—
Anger	0.240	0.229	<0.001	0.07 ^*^
Negative	0.450	0.465	>0.001	—
Sadness	0.202	0.229	<0.001	0.21 ^*^
Joy	0.249	0.317	<0.001	0.38 ^**^
Surprise	0.136	0.152	>0.001	—
Disgust	0.195	0.198	>0.001	—

Size effects (r): ^*^Small difference (0.1≤r<0.3). ^**^Medium difference (0.3≤r<0.5). ^***^Large difference (0.5≤r<0.7). ^****^Very large difference (r≥0.7) [53].

### 3.3. Sentiment Strengths of NMPDU Tweets

**Figure 2:**
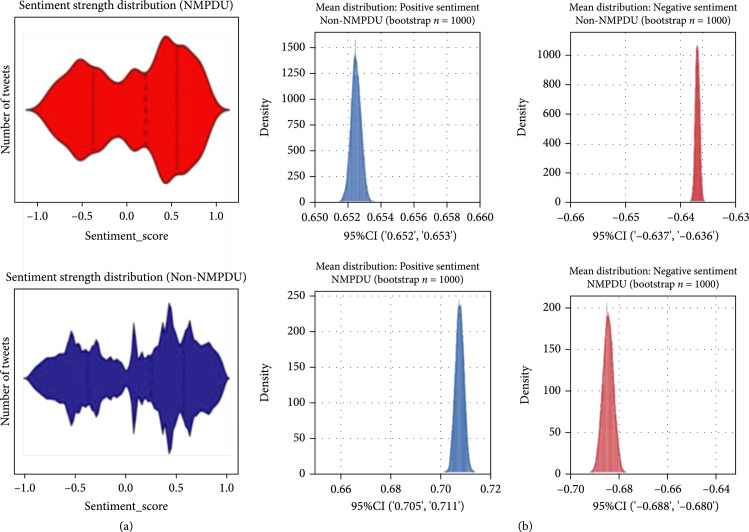
(a) Sentiment strength distributions for NMPDU and non-NMPDU posts. (b) Comparison of the means and confidence intervals of extreme positive tweets (sentiment score>0.5) and negative tweets (sentiment score<−−0.5) in both non-NMPDU and NMPDU categories.

We intended to measure and compare the sentiment polarities and strengths between the NMPDU and non-NMPDU tweets from the same users. As illustrated in Figure 2(a), the NMPDU tweets contain larger magnitudes of extreme positive and negative sentiments (tweets with a positive score of >0.5 or a negative score of < −0.5) compared to the non-NMPDU tweets. We empirically compared the sentiment strength means and confidence intervals for highly polarized tweets (sentiment<−0.5 or > 0.5) from the NMPDU and non-NMPDU categories Figure 2(b). The sentiment strength means of the NMPDU tweets (both positive 95% CI [0.705, 0.711] and negative tweets 95% CI [-0.688, -0.680]) are higher in magnitude than the non-NMPDU tweets (positive 95% CI [0.652, 0.653] and negative tweets 95% CI [-0.637, -0.636]). The highly polarized nature of the NMPDU tweets indicates potential emotional triggers associated with NMPDU behavior. 

### 3.4. Personal and Social Concern Analysis

We measured differences between the tweets from the NMPDU and non-NMPDU groups in terms of the following content dimensions: personal concern (e.g., work, leisure, home, money, religion, and death), social content (e.g., family and friends), time orientation content (e.g., past focus), core drive content (e.g., achievement), and biological process content (e.g., health and body). Table 4 presents the medians, Mann–Whitney U test results, and the effect sizes of the comparisons between the NMPDU group and the control group tweets. In addition, we present comparisons between the groups for each medication category (i.e., opioids, benzodiazepines, stimulants, and polysubstance) in Supplementary S.4. 

**Table 4:** Comparison of the personal and social concern content between the users from the NMPDU and control groups.

Content	Variables	Median: NMPDU group	Median: control group	Different (NMPDU versus control) Mann–Whitney U test results	
				p value (p)	Effect sizes (r)
Social content	Family	0.0333	0.025	<0.001	0.19 ^*^
	Friend	0.0187	0.020	>0.001	—

Time orientation content	Past focus	0.241	0.140	<0.001	0.59 ^***^

Core drive content	Achieve	0.098	0.204	<0.001	0.63 ^***^

Biological process content	Health	0.047	0.056	<0.001	0.15 ^*^
	Body	0.112	0.086	<0.001	0.28 ^*^

Personal concern content	Work	0.098	0.215	<0.001	0.60 ^***^
	Leisure	0.120	0.272	<0.001	0.71 ^****^
	Home	0.031	0.058	<0.001	0.50 ^***^
	Money	0.055	0.086	<0.001	0.41 ^**^
	Religion	0.032	0.041	<0.001	0.23 ^*^
	Death	0.018	0.016	>0.001	—

Size effects (r): ^*^Small difference (0.1≤r<0.3). ^**^Medium difference (0.3≤r<0.5). ^***^Large difference (0.5≤r<0.7). ^****^Very large difference (r≥0.7) [53].

The users from the NMPDU group express significantly more social content related to family (p<0.001, r=0.19) than the users from the non-NMPDU group, but no significant difference is observed between the groups in content related to friends (p>0.001) (Table 4). The comparisons in the personal concern content demonstrate that the users from NMPDU group express significantly less personal concern content related to work (p<0.001, r=0.60), leisure (p<0.001, r=0.71), home (p<0.001, r=0.50), money (p<0.001, r=0.41), and religion (p<0.001, r=0.23) compared to the users from the control group. No significant difference is found in the death variable (p>0.001). For biological process content, the users from the NMPDU group tend to use less content related to health (p<0.001, r=0.15) and use more content related to the body (p<0.001, r=0.28) than the users from the control group. Comparing both groups based on time orientation content shows that the users from the NMPDU group tend to discuss significantly more content related to the past (p<0.001, r=0.59) than the users from the control group. Finally, the users from the NMPDU group express significantly less core drive content related to achievement (p<0.001, r=0.63) compared to the users from the control group. 

#### 3.4.1. Gender Differences in Concerns within the NMPDU Group

Females within the NMPDU group express significantly more social content related to family (p<0.001,r=0.27) and friends (p>0.001,r=0.50) compared to the male NMPDU users (Table 5 and Figure 1). No significant gender differences are observed in personal concern content related to all but the home variable, with female NMPDU users expressing more content related to home (p>0.001,r=0.41) compared to the male NMPDU users. For biological process content, the female NMPDU users tend to use more content related to health (p<0.001,r=0.37), while no significant gender difference exists in content related to the body. For time orientation content, the female NMPDU users tend to discuss significantly more content related to the past (p<0.001,r=0.21) than the male NMPDU users. Finally, there is no significant gender difference in core drive content related to achievement.

**Table 5:** Comparison of the personal and social concern content between the male and female users from the NMPDU group.

Content	Variables	Median: male NMPDU group	Median: female NMPDU group	Different (male NMPDU versus female NMPDU l) Mann–Whitney U test results	
				p value (p)	Effect sizes (r)
Social Content	Family	0.029	0.038	<0.001	0.27 ^*^
	Friend	0.013	0.026	<0.001	0.50 ^***^

Time orientation Content	Past focus	0.229	0.267	<0.001	0.21 ^*^

Core drive content	Achieve	0.105	0.114	>0.001	—

Biological process Content	Health	0.041	0.057	<0.001	0.37 ^**^
	Body	0.108	0.120	>0.001	—

Personal concern Content	Work	0.092	0.110	>0.001	—
	Leisure	0.121	0.125	>0.001	—
	Home	0.025	0.040	<0.001	0.41 ^**^
	Money	0.056	0.056	>0.001	—
	Religion	0.033	0.033	>0.001	—
	Death	0.018	0.018	>0.001	—

Size effects (r): ^*^Small difference (0.1≤r<0.3). ^**^Medium difference (0.3≤r<0.5). ^***^Large difference (0.5≤r<0.7). ^****^Very large difference (r≥0.7) [53].

### 3.5. Potential Reasons for NMPDU

Table 6 shows the summary of the potential reasons for NMPDU and frequently used keywords indicating these reasons for each medication category. We interpreted the identified topics and selected potential reasons. These reasons were inferred by manually inspecting the frequent words within each category qualitatively, guided by the National Survey on Drug Use and Health (NSDUH) surveys [51]. 

**Table 6:** Potential reasons for NMPDU extracted from topic modeling content analysis.

Potential reasons Categories	Potential reasons	Frequent words across detected topics in each category
Opioid	To relieve physical pain	Pain, hospital, nauseous, and surgery
	To get high or use it with illicit drug	High, dope, and heroin
	To help with sleep	Sleep
	To help with emotions (stress)	Stress
	Hooked and addiction	Hook and addiction
	To relax	Relax, cool, recreational, and good
	To use it with smoking	Smoking, blunt, and weed
	To use it with alcohol	Drunk, liquor, wine, whiskey, and vodka

Stimulants	To help study	Finals, college, semester, study, school, exam, writing, help, test, homework, and essay
	To stay awake	Awake, espresso, and caffeine
	To use it with alcohol	Drank, wine, and whiskey
	Hooked and addiction	Hook and addiction
	To use it with smoking	Smoking and weed

Benzodiazepines	To get high or use it with illicit drug	High and cocaine
	To help with sleep	Sleep, asleep, and slept
	To help with emotions	Anxiety
	Hooked and addiction	Addiction
	To relax	Relax, cool, recreational, and happy
	To use it with smoking	Smoking, marijuana, blunt, and weed
	To use it with alcohol	Drank, vodka, tequila, liquor, wine, and whiskey

Polysubstance	To help study	Finals and study
	To get high or use it with illicit drug	High, heroin, and cocaine
	To help with emotions	Ecstasy and satisfaction
	Hooked and addiction	Addiction and addict
	To socialize	Birthday, couple, and friends
	To use it with smoking	Cigarettes, blunt, and weed
	To use it with alcohol	Drunk, wine, whiskey, and vodka
	To help with sleep	Sleep

## 4. Discussion

Our emotion analysis showed significant differences in the emotion-indicating expressions of the tweets between users from the NMPDU and control groups. Relative to users from the non-NMPDU group, users from the NMPDU group posted more emotionally negative content and less emotionally positive content in their Twitter posts. Relative to the non-NMPDU tweets, the NMPDU tweets contained higher numbers of extremely polarized (positive or negative) tweets, indicating possible emotional triggers associated with NMPDU. We also found significant differences in the contents shared between female and male nonmedical users of prescription drugs. Compared to female users, male users expressed higher anger and lower positivity, joy, anticipation, and sadness in their posted contents. In terms of social and personal content, compared to the male users, female users shared more content related to social life (friends and family), health, and personal concern (home). Interestingly, while there were unique and detectable differences in the contents between male and female nonmedical prescription drug users, the differences were consistent across different drug categories. These findings perhaps indicate that the underlying reasons behind NMPDU may be associated with cohort-level behavioral characteristics more than the properties of the substances themselves. From the perspective of public health, the insights obtained through this large-scale analysis of social media data may help customize awareness and intervention programs to targeted cohorts in order to mitigate the population-level impacts of NMPDU.

Our study adds to the growing body of literature focusing on the intersection of substance use and behavioral health. The findings from our large-scale social media analyses are consistent with previous results from a survey-based study [54] that showed that those who reported specific feelings, such as hopelessness, sadness, or depression, are more likely to report nonmedical use of opioids, stimulants, sedatives, and antidepressants. The consistency in findings across studies demonstrates the utility of social media for NMPDU surveillance—in this case, surveillance may not only help estimate NMPDU at the population level but also provide in-depth insights into the emotional and behavioral drivers of NMPDU. Social media-based surveillance systems have the potential of operating in close to real time while costing less than traditional surveillance systems and have the ability to include seldom heard populations (e.g., people without health coverage in the US). While social media-based surveillance systems will not replace the traditional ones, they may offer complementary information.

A previous study reported an association between the uses of emotional words (user-generated natural language) and individuals’ experiences (individual differences in mood, personality, and physical and emotional well-being) [26]. The study showed that negative emotion words were associated with psychological distress and poor physical health, whereas higher positive emotion words are associated with better well-being and physical health. Thus, although our study did not directly examine such an association among the NMPDU users on Twitter, we posit that the higher numbers of negative emotion words of the users from the NMPDU group are likely associated with greater psychological distress and poorer physical health compared to their non-NMPDU counterparts, a hypothesis that we plan to study in future work.

Our study also demonstrates that potential specific reasons behind NMPDU may be derived from social media data, and this finding may have major public health implications. This information can be useful to policymakers for implementing measures for drug use prevention, intervention, and treatment in their communities [51]. As shown in Table 6 and elaborated in Supplementary material S.5, “to relieve pain” is one reason for the NMPDU of opioids, indicating that opioids are often used for treating pain and that not all prescription opioid use is for recreational reasons or due to addiction. The reason “to help with emotions” is common in the NMPDU of opioids and benzodiazepines, suggesting that these two categories of medications are potentially used nonmedically for coping with emotional problems. “To help with sleep” is reported as a reason for the NMPDU of opioids, benzodiazepines, and polysubstance, suggesting that many people nonmedically use these substances for addressing their sleep problems. Over the recent years, the coingestion of opioids with benzodiazepines has led to rising overdose-related deaths [55]. Since our findings indicate that many people may be using these substances for addressing sleep problems, more efforts are called for to educate the general public about nonpharmacological, safer strategies to mitigate sleep problems/improve sleep quality. Healthcare providers could help identify and intervene with the root causes of their patients’ sleep problems. These efforts could contribute to reducing drug overdose-related mortality. The topic analysis also suggests the nonmedical use of stimulants is often to enhance educational performance and for staying awake. Past research has shown that nonmedical use of prescription stimulants, such as Adderall®, is widespread among college students [56, 57], and our findings agree with these studies. Overdose deaths due to stimulants (prescription and illicit, particularly co-use with fentanyl and other opioids) are rapidly increasing in the US, which might be partly attributed to the many years of widespread prescription stimulant use in educational settings [58]. Students could benefit from awareness programs in educational institutions or adolescent/young adult healthcare settings to prevent adverse, often fatal, health consequences caused by stimulant use. The topics associated with all the medication categories are indicative of co-use of prescription drugs with other legal substances such as alcohol and tobacco and indicative of NMPDU due to substance use disorder. Specifically for opioids, benzodiazepines, and polysubstances, there are topics that are indicative of co-use with illicit substances such as cocaine and heroin. Topics associated with nonmedical use of benzodiazepines are indicative of their use for relieving stress. Finally, topics associated with polysubstances are indicative of their use in social settings.

Substance use and its impacts are not evenly distributed among males and females. In terms of alcohol and illicit drug use, men of ages 12 and older report higher usage rates than women [1, 2]. While women have lower rates of alcohol and substance use, they are more likely to have a serious mental illness than men [59, 60]. Research shows that women are more likely to be diagnosed with anxiety or depression (including postpartum depression), and men are more likely to have substance use or antisocial disorders [61]. In terms of death rates, men are substantially more likely to die from substance overdoses than women [62]. Our past study has shown that women and men report nonmedical use of prescription stimulants and benzodiazepines at similar rates over social media, but more men report nonmedical use of prescription opioids [36]. To the best of our knowledge, this is the first study that utilizes large-scale social media data to study the gender-specific distribution of sentiments and emotions associated with NMPDU.

### 4.1. Limitations

Our study has several limitations. A major limitation is that data from social media may not be well representative of the overall population. Social media users tend to be younger and technologically savvy, resulting in a biased sample. However, it is also unlikely that any other resource matches the scale and reach of social media, and as the demographics shift, more and more older adults are reachable via social media [63]. As mentioned above, the triangulation of social media and traditional survey data (or any other offline data source) to study NMPDU can help minimize the potential biases in the representative samples. There are also limitations associated with the methods we employed. We applied topic modeling to discover potential reasons for NMPDU. Unlike supervised methods (e.g., classification), which can be evaluated against human experts, it is not possible to thoroughly evaluate the performance of topic modeling. The performance of topic modeling may vary, and there is no mechanism to evaluate such approaches in a task-oriented manner. Also, our study findings are dependent on the classification performances of the machine-learning and NLP pipelines. The performances of these methods are not 100% accurate and may add further biases in the downstream analyses. 

Social media data might contain bias influenced by social norms, culture, and expectations. Identifying and controlling such factors while designing the study is challenging due to the nature of social media data and the need to preserve users’ privacy. Social media-based systems can potentially be integrated with traditional data sources (e.g., survey data) to obtain a more complete picture of population-level patterns associated with substance use.

One major limitation of the gender-wise analysis is that the gender classifier is developed under the assumption of a binary gender system (i.e., male and female). Although this setup accommodates the majority of the population, it excludes the nonbinary population, who often do not receive the necessary research attention and effort. The future directions to overcome this limitation include updating the classifier to accommodate nonbinary population or directly collecting data from self-identified nonbinary persons upon their approval for inclusion.

Our study only includes publicly available user profiles’ data from Twitter. Therefore, private profiles or information that are missed or not posted by the users are not represented in this study, and consequently, this study may not be fully representative of the overall population of social media users. However, since this study includes large-scale data analyses, we believe the use of such extensive data may help generalize the overall results of this study.

This study did not include demographic data other than gender (binary), such as age, race, ethnicity, and social class. We plan to study these demographic factors in detail in our future studies, as more advanced methods for automatically detecting these factors are developed. This study also did not include geographical location data of the users to study the geographic distribution of NMPDU. We plan to include this information in our future work.

LDA best learns descriptive topics [64], and the performance of LDA has several limitations that need to be considered while conducting a practical experiment. Generally, LDA model outcomes vary with the changing of the hyperparameters, making it difficult to determine its effectiveness without thorough empirical reviews [65]. A large dataset is typically the most important requirement as it is theoretically impossible to identify topics from a small number of data. The lengths of documents also play a vital role: unsatisfactory performance of the LDA is likely if documents are too short; therefore, using LDA for topic identification from tweets requires, in many cases, the combining of several tweets from the same class to create a large document. Likewise, the number of topics should not be too large as the interpretation may become inescapably inefficient [65]. 

## 5. Conclusion

Social media provides a unique opportunity to study NMPDU at a macrolevel, unobtrusively, and in close to real time. Although social media data presents its own challenges, such as the use of colloquial expressions and nonstandard spelling variants, advances in machine learning and NLP methods have enabled us to leverage the vast knowledge encapsulated in this resource. Our study identified important significant differences in the texts associated with NMPDU and non-NMPDU users, and also between males and females within the NMPDU group. The current study has a number of limitations primarily associated with the data source (i.e., social media) and the methods applied to characterize the data. Future work should address these limitations to improve social media-based surveillance of substance use.

## Data Availability

The data used in this study are publicly available from Twitter. However, it cannot be distributed by the authors. Statistical data extracted from the Twitter content reported in this paper’s findings and the source code needed to replicate the findings can be downloaded from the following code link: https://drive.google.com/file/d/1udz4p5lIuVwHkhicYPAcex55YH2WmHbU/view?usp=sharing. The authors can provide the researchers with the IDs required for downloading tweets directly from the Twitter application programming interface upon reasonable request. Additional data and information are available from the authors upon reasonable request.

## Supplementary Materials

Supplementary Materials Supplementary 1: definition and terminology, Supplementary 2: data collections, Supplementary 2.1: keywords list, Supplementary 3: Normality and statistical tests, Supplementary 4: difference between each NMPDU category and non-NMPDU (control group) (Emotional analysis and personal and social concern analysis), and Supplementary 5: topic modeling.
